# Variability of Skin Pharmacokinetic Data: Insights from a Topical Bioequivalence Study Using Dermal Open Flow Microperfusion

**DOI:** 10.1007/s11095-020-02920-x

**Published:** 2020-09-28

**Authors:** Manfred Bodenlenz, Thomas Augustin, Thomas Birngruber, Katrin I. Tiffner, Beate Boulgaropoulos, Simon Schwingenschuh, Sam G. Raney, Elena Rantou, Frank Sinner

**Affiliations:** 1 HEALTH - Institute for Biomedicine and Health Sciences, Joanneum Research Forschungsgesellschaft m.b.H, Neue Stiftingtalstrasse 2, 8010 Graz, Austria; 2 Division of Endocrinology and Diabetology, Department of Internal Medicine, Medical University of Graz, Auenbruggerplatz 15, 8036 Graz, Austria; 3 Division of Therapeutic Performance Office of Research and Standards Office of Generic Drugs, United States (U.S.) Food and Drug Administration, 10903 New Hampshire Avenue, MD 20993 Silver Spring, USA; 4 Division of Biostatistics VIII, Office of Biostatistics, Office of Translational Sciences, United States (U.S.) Food and Drug Administration, 10903 New Hampshire Avenue, MD 20993 Silver Spring, USA

**Keywords:** Topical bioequivalence, inter- and intra-subject variability, dermal open flow microperfusion, microdialysis, acyclovir, skin pharmacokinetics

## Abstract

**Purpose:**

Dermal open flow microperfusion (dOFM) has previously demonstrated its utility to assess the bioequivalence (BE) of topical drug products in a clinical study. We aimed to characterize the sources of variability in the dermal pharmacokinetic data from that study.

**Methods:**

Exploratory statistical analyses were performed with multivariate data from a clinical dOFM-study in 20 healthy adults evaluating the BE, or lack thereof, of Austrian test (T) and U.S. reference (R) acyclovir cream, 5% products.

**Results:**

The overall variability of logAUC values (CV: 39% for R and 45% for T) was dominated by inter-subject variability (R: 82%, T: 91%) which correlated best with the subject’s skin conductance. Intra-subject variability was 18% (R) and 9% (T) of the overall variability; skin treatment sites or methodological factors did not significantly contribute to that variability.

**Conclusions:**

Inter-subject variability was the major component of overall variability for acyclovir, and treatment site location did not significantly influence intra-subject variability. These results support a dOFM BE study design with T and R products assessed simultaneously on the same subject, where T and R treatment sites do not necessarily need to be next to each other. Localized variation in skin microstructure may be primarily responsible for intra-subject variability.

## Introduction

A considerable amount of research has been carried out in recent years to promote new sensitive and discriminating methods for the BE assessment of topical dermatological drug products based on pharmacokinetic (PK) endpoints, among them skin stripping (tape stripping), dermal microdialysis (dMD) and dermal open flow microperfusion (dOFM) [1–6]. Although such methods for topical in vivo permeation studies are promising for BE assessments, the resulting data are highly variable - like most of topical PK data. Therefore, the sources of this variability and their potential impact on the outcome of BE assessments remain a subject of major interest.

Characterization of data variability has been performed previously with clinical dMD data in studies on topically applied drugs [1, 7, 8]. Overall variabilities from 40 to 61% have been observed in vivo [1, 7], and differences in skin barrier function between subjects have been assumed to be the major contributor to overall variabilities in topical drug penetration [1, 8]. Several dMD studies have also investigated intra-subject data variability [1, 9–12], and they have largely attributed this intra-subject variability to biological differences between the individual treatment sites [1, 13]. However, results have certain limitations as they are derived mostly from studies on highly penetrating topical drug products performed with limited probe numbers.

As part of a U.S. Food and Drug Administration (FDA) funded collaborative research effort to evaluate PK-based methods for topical BE assessment, we have recently performed a clinical dOFM study to assess the BE of commercially available acyclovir cream, 5% products [2]. This study included 20 subjects with six treatment sites per subject and two dOFM probes per treatment site; it delivered a comprehensive data set that verified the topical BE of the reference product (R) to itself and identified the test product (T) as being non-bioequivalent to the R product. During this study, skin barrier properties were assessed, demographic data recorded, and methodological factors monitored. The resulting data set included data on the dermal PK and on multivariate biological-methodological parameters that might potentially have been associated with the observed variability, thus representing an ideal data set with which to investigate skin PK data variability after topical drug application, and with which to evaluate the sources of that variability.

We therefore aimed to characterize the sources of variability in the dermal PK data using exploratory statistical analyses with this extensive set of data. An understanding of the mechanistic basis for variability in such studies, and the implications for controlling the variability and minimizing the impact of variability on the sensitivity of BE assessments, is essential in order to optimize dOFM, dMD, and potentially other topical BE study designs, as well as to support reliable power calculations for these clinical BE studies.

## Material and Methods

### Clinical dOFM BE study

The clinical dOFM study included 20 human subjects (Caucasian, age 28 ± 5 years, seven female) treated with acyclovir cream, 5% [2]. In brief, the study design included 6 treatment sites per subject (3 treatment sites on each thigh) and 2 dOFM probes per treatment site. R and T were applied in a randomized order of either R–R–T or T–R–R on each thigh. R was applied twice on each thigh to evaluate the reproducibility of the dOFM data and to serve as positive control for BE (R vs R). T served as negative control and was compared to the R treatment in the center of the test triad (T vs R). The dOFM probes were inserted intradermally and probe depth was assessed by longitudinal ultrasound scanning (GE LOGIQ e R6 device with linear 22 MHz probe; GE Healthcare, Vienna, Austria) after sampling. Dermal interstitial fluid was continuously sampled with a flow rate of 1 μL/min using sterile perfusate (physiological saline containing 1% albumin and 600 mg/dL glucose) from 1 h pre-dose to 36 h post-dose, with post-dose sampling intervals of 4 h. Before dosing, the skin temperature of each of the 6 treatment sites was measured (Infrared thermometer TDT8806, Thomson Health Care, France) and transepidermal water loss (TEWL) was measured in duplicate on each thigh (TEWL; Aquaflux AF200; Biox Ltd., London, UK). Skin impedance measurements were performed with a 3-electrode setup in a frequency range from 1 to 100 Hz [14], and skin conductance at 100 Hz was used to describe the individual skin barrier property.

Acyclovir cream, 5% was applied in a homogenous layer (15 mg cream/cm^2^) to each respective treatment site following a standardized procedure. Thereafter the treatment site was protected by a non-occlusive transparent shield over a duration of 36 h post-dose [2]. The dose of 15 mg cream/cm^2^ took into consideration the low permeation of acyclovir and was selected based on results from pilot studies. Room temperature and relative humidity were tightly controlled throughout the experiment (22 ± 1°C, 40–60% relative humidity). Glucose and lactate concentrations in the dOFM samples were measured at the bedside (Super GL; Dr. Müller Gerätebau GmbH, Freital, Germany) as indicators to roughly estimate the stability of the relative recovery, which are straightforward to evaluate at the bedside in each of the 2400 samples. Acyclovir was measured from frozen samples as previously described [2]_._

### Data Set and BE Evaluation Results

The data set from the clinical dOFM BE study [2] included data on the dermal PK of acyclovir delivered topically from the R and T products parameterized as PK endpoint data (area under the dermal concentration-time curve (AUC) and peak/maximum dermal concentration (C_max_) values), as well as demographic data, data on each subject’s skin barrier properties (TEWL, skin conductance), skin temperature, dOFM probe-related data (probe depths), and data derived from methodological monitoring (glucose-loss, recovered lactate and sample mass) (Table 1).

**Table 1:** Data set of the acyclovir dOFM BE study

Type of data	Subjects	x legs	x sites	x probes	= total
Subjects demographic data (sex, age, BMI)	20				20
Conductance, TEWL, skin temperature at t = 0 h	20	2			40
Topical treatment sites with drug application (R, T)	20	2	3		120 treatment sites
dosing 15 mg/cm^2^ of R	20	2	2		80 treatment sites for R
dosing 15 mg/cm^2^ of T	20	2	1		40 treatment sites for T
dOFM probes inserted in topical treatment sites (R, T)	20	2	3	2	240 probes
Probe depths for all dOFM probes at t = 36 h (R, T)	20	2	3	2	240 probe depths
dOFM acyclovir profiles, AUCs, C_max_ for R	20	2	2	2	160 (1600 samples)
incl. Glucose-loss, volume profiles from −1 to 36 h^1^	20	2	2	2	160 (1600 samples)
dOFM acyclovir profiles, AUCs, C_max_ for T	20	2	1	2	80 (800 samples)
incl. Glucose-loss, volume profiles from −1 to 36 h^1^	20	2	1	2	80 (800 samples)
dOFM sampling hours (37 h per probe) ^1^	20	2	3	2	8880 h

^1^37 h of sampling: One hour baseline sampling followed by 36 h of post-dose sampling in 4 h- intervals (10 samples per probe).

The results of the BE evaluation of this study have been published by Bodenlenz et al. [2]. The relative bioavailability of R vs. R and T vs. R has been evaluated based on the conventional BE PK endpoints, AUC and C_max_ in the dermis, where the criterion for establishing the BE of a T to an R is that the 90% confidence interval of the geometric mean ratio between the T and R falls within 0.80 and 1.25. In brief, the positive control products (R vs. R) were accurately and reproducibly confirmed to be bioequivalent [AUC0–36 h (0.86–1.18) and C_max_ (0.86–1.21)], while the negative control products (T vs. R) were sensitively discriminated not to be bioequivalent for both parameters [AUC0–36 h (0.69–1.05) and C_max_ (0.61–1.02)].

### Statistical Analysis

We performed exploratory statistical analyses of the dOFM data set (Table 1). Data normality was tested with the Kolmogorov-Smirnov test. The overall variability of the dermal endpoint parameter logAUC was expressed as the % coefficient of variation (CV). Its main components, the inter- and intra-subject variability, were determined by performing analyses of variance (ANOVAs) with the fixed factors subject, treatment site and probe. We based our analysis on AUC/logAUC because this PK parameter incorporates multiple data points, making it not only information-rich and well described by the underlying skin permeation data but also relatively robust, and therefore, most suitable for the identification of the sources of variability. Moreover, and independently, we focused our analysis on the variability of R, as the higher number of replicates for R facilitated such analysis.

The sources of inter-subject variability were identified using multiple linear regression with a backward elimination technique and the Pearson’s product-moment correlation. The following variables were analyzed: sex, age, body mass index (BMI), transepidermal water loss (TEWL), skin conductance, skin temperature (pre-dose), dOFM probe depths after sampling, dOFM sample volumes, and exchange rates of glucose and lactate.

The relevant sources of intra-subject variability were assessed using multiple linear regression and descriptive statistics. To determine whether intra-subject AUC values were normally distributed, we analyzed the distribution within each subject separately, and within the aggregated data for all subjects based on the “normalized” variables (X-μ)/σ, where X is the original (untransformed) variable, μ is the intra-subject mean, and σ the intra-subject standard deviation: X is normally distributed within subjects if the “normalized” variable (X-μ)/σ is normally distributed. Statistics was performed using SAS and plots were created using Origin Pro 2018G.

## Results and Discussion

### Overall variability

**Fig. 1:**
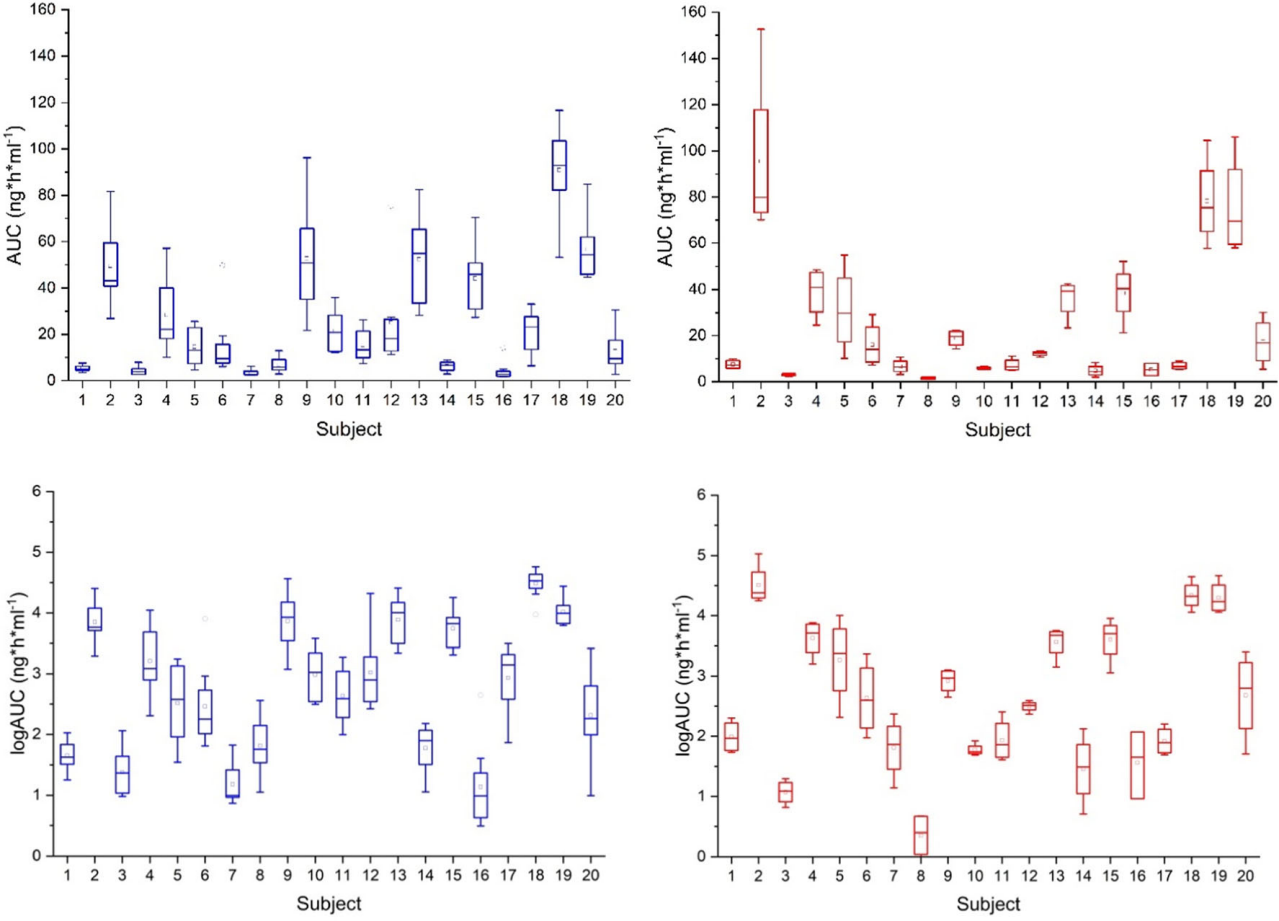
AUC values (0–36 h) of all 240 probes in 20 subjects. Top panels: Untransformed AUC values for R (8 probes per subject, left side) and T (4 probes per subject, right side). Bottom panels: Log-transformed AUC values for R (left side) and T (right side)

The AUC values showed considerable differences among the 20 subjects and considerable intra-subject variability (Fig. 1, top panels). After log transformation of the AUC values (Fig. 1, bottom panels), the overall variability (CV) of the dermal endpoint parameter logAUC was 39% for R and 45% for T. This is in agreement with the lower range of variabilities observed in previously performed dMD studies (CV ranging from 42 to 93%) [11].

**Fig. 2:**
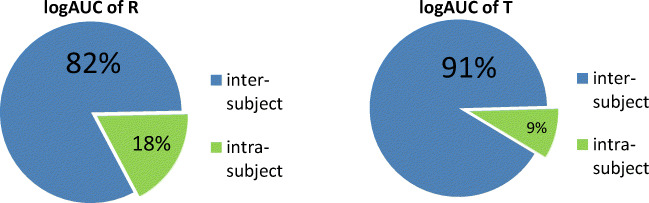
Main sources of variability for R (left side) and T (right side) derived from an ANOVA

An ANOVA of the logAUC values showed that the inter-subject variability was the major source of variability (Fig. 2). The remaining 18% (R) and 9% (T) were attributed to intra-subject variability (Fig. 2).

Our ANOVA results showed inter-subject variability to be the greatest source of variability, with a much smaller proportion of the variability arising from intra-subject variability; the intra-subject variability in our study was similarly low or lower compared to the two previously published dMD studies [1, 12]. The dMD study on topical lidocaine products in 8 subjects attributed 61% of the overall variability to the inter-subject-variability and 39% to intra-subject variability based on an ANOVA analysis [1]. The dMD study on topical ketoprofen in 18 subjects did not calculate the relative contributions of inter- and intra-subject variability based on an ANOVA analysis, but the reported CVs suggest an inter-subject variability of approximately 85% and an intra-subject variability of approximately 15%, i.e. similar to our study [12]. The dominant contribution of inter-subject variability to overall variability has also been reported in studies using tape stripping for the purpose of topical BE assessment [4]. The low intra-subject dOFM variability found in our study indicates a relatively minor influence of any localized variations in skin permeation on overall variability as well as a relatively low contribution of methodological factors to overall variability, which we attribute to the extensive optimization and standardization of the dOFM materials and study procedures. Intra-subject variability includes the factors *site* and *probe*, and is the sum of the variations caused by localized variations in skin as well as methodological variations. The ANOVA performed by Benfeldt et al. found equal contributions to variability by the factors *site* and *probe* [1]. However, we refrained from discriminating between these two factors because they are interlinked and discrimination by ANOVA might yield misleading results. Pinnagoda et al. performed an ANOVA of TEWL data which indicated that inter-subject differences contributed between approximately 79% to 92% (depending on outlier treatment) of the overall variability, while intra-subject differences contributed between approximately 8% to 21% of the overall variability [15]. These high inter-subject differences explain, in part, the relatively large numbers of subjects typically required to adequately power comparative clinical endpoint BE studies for topical dermatological drug products.

### Inter-Subject variability

**Fig. 3:**
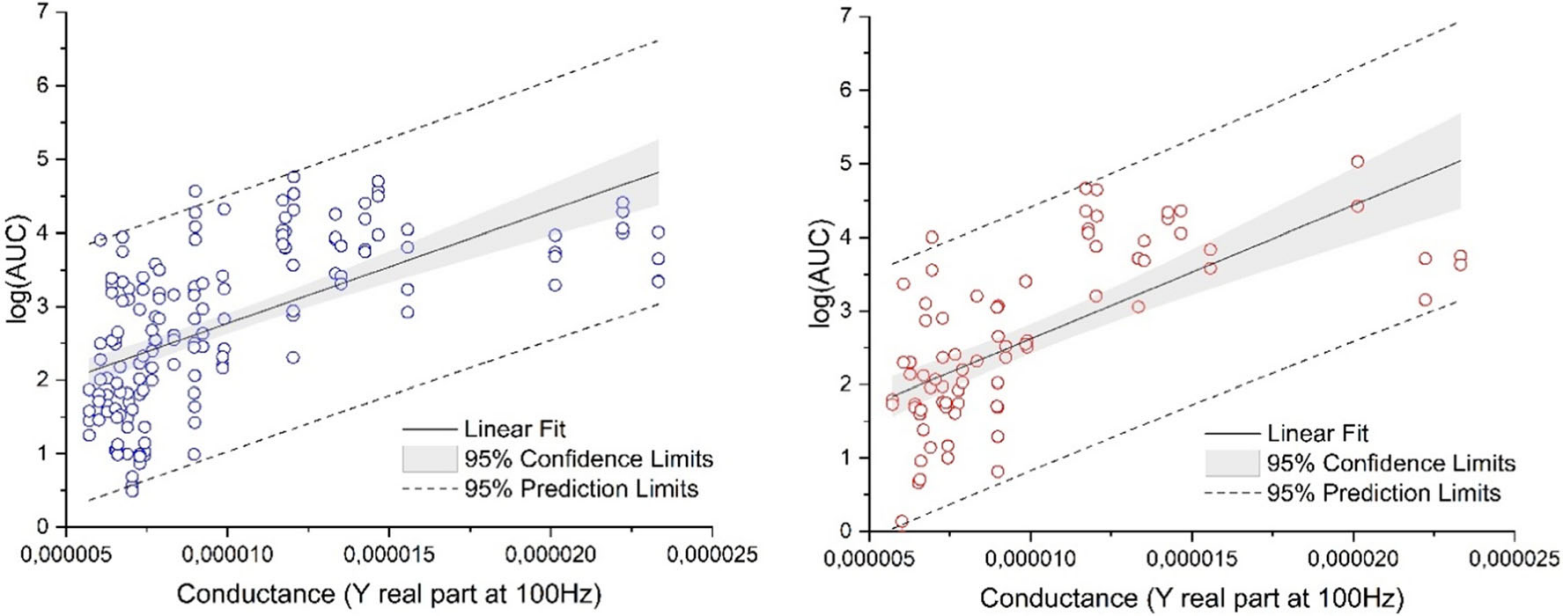
Multiple linear regression analysis combined with a backward elimination technique identified skin conductance as the sole parameter among those evaluated that appeared to be consistently associated with inter-subject variability. Left side: Relationship of logAUC vs. conductance for R, right side: Relationship of logAUC vs. conductance for T

To characterize the skin barrier properties of the subjects, we measured TEWL, skin conductance and skin temperature (pre-dose). Furthermore, dOFM-related parameters such as sample volumes (flow rate), exchange rates of glucose (loss from perfusate) and exchange rates of lactate (gain from interstitial fluid) were assessed during dOFM sampling. In addition, dOFM probe depths were measured after sampling. Demographic variables (sex, age, BMI) were also analyzed. The parameters were examined in a multiple linear regression analysis with a backward elimination technique. The regression model started with all above mentioned parameters. For the R product, 39% of the logAUC-variation could be explained by two parameters, skin conductance (*p* < 0.0001) and lactate (*p* = 0.0389). For the T product, 44% of the logAUC-variation could be explained by the skin conductance parameter alone (p < 0.0001); the skin conductance parameter was the only parameter consistently associated with logAUC for both, R and T products (Fig. 3).

Pearson’s product-moment correlation identified a close relationship between the logAUC values and the individual skin conductance (r = 0.65, *p* < 0.001). The relationship between TEWL and logAUC values was weaker (R: r = 0.31, *p* = 0.054; T: r = 0.37, *p* = 0.017). Conductance and TEWL values correlated well with each other for R (r = 0.72, *p* < 0.0001). A possible explanation for the difference in correlation between conductance and TEWL values to the logAUC values might be that results from conductance are more sensitive to the state of hydration of the SC [16]. Increase in hydration of the SC leads to a higher permeability of the SC to topically applied substances [17]. Subjects with a more hydrated SC may therefore have increased AUC values and also increased conductance values. TEWL values may not be elevated to the same extent.

The skin temperature varied from 28.1°C to 34.0°C between subjects, and also varied slightly between the intra-subject treatment sites. The skin temperature showed a small but statistically significant positive correlation with logAUC values (r = 0.21, *p* = 0.008). Skin temperature depends to a certain degree on physiological processes in the viable layers of the skin, such as temperature regulation by skin capillary perfusion (e.g. dilation or constriction) and the rate of local blood flow. Independent of these underlying mechanisms, the skin temperature itself may have the potential to directly affect the rate of drug release from a topically applied formulation, potentially by influencing the drying rate (metamorphosis of the product on the skin) as well as the drug partitioning and/or diffusion into the skin. However, skin temperature did not significantly differ between subjects and explained only approximately 4% of the logAUC-variation and was thus not considered to be a main cause of inter-subject variability in our study.

The mean lactate concentrations in the dOFM samples varied between subjects from 6.7 to 10.4 mg/dL and showed a small but statistically significant positive correlation to the logAUC values for R (r = 0.26, *p* = 0.0012). For the smaller data set of T this correlation was not identified. This correlation could be explained by the fact that the lactate concentration in the dOFM sample (at least partially) reflects the *relative recovery* of molecules from the surrounding of the dOFM probe, which links the recovered lactate to the recovered acyclovir concentration. The correlation of lactate concentrations to the logAUC values was statistically significant, but rather low, which indicates a small contribution of the factor *relative recovery* to the inter-subject variability. Such a correlation should be explained by the fact that the relative recovery depends on the interstitial fluid content of the tissue, which slightly varies with the individual hydration status of the subject. Lactate is also a descriptor of local metabolism and trauma, which has already been well characterized by others for the use of sampling probes [18]. None of the other dOFM probe-related parameters (probe depth, sample volume, glucose concentration; all *p* > 0.10) showed any correlation with the logAUC values.

These results are consistent with results from a lidocaine dMD study that analyzed a number of potential co-variates and observed no correlation between factors like probe depth, room temperature, or humidity and topical drug kinetics [1]. Also Stagni et al. did not find any correlation between the kinetics of dermal drug absorption and probe depth in a dMD study investigating iontophoretically delivered propranolol [13]. However, the apparent lack of a correlation between probe depths and logAUC values in our acyclovir dOFM data set is in contrast to results from a dOFM study with the highly lipophilic drug clobetasol-17-propionate, where minor probe depth differences of 0.2 mm were shown to have an apparent influence on the observed AUC values [19].

### Intra-Subject variability

**Fig. 4:**
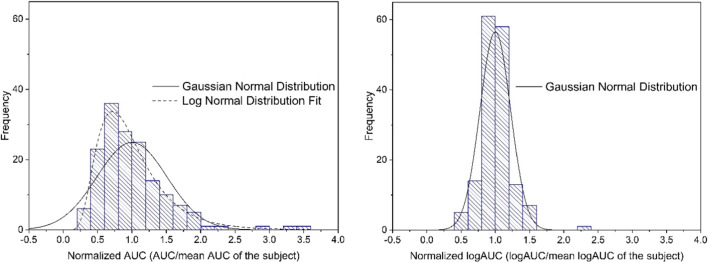
Distribution of the aggregated normalized intra-subject AUC values for R (0–36 h), Left side: Normalized AUC values. Right side: Normalized logAUC values

The AUC values of the 8 R probes showed a positively skewed distribution in 17 of 20 subjects (mean skewness: 0.88 ± 0.93, range: −0.82 to 2.50). The AUC values normalized based on the individual means (aggregated normalized AUC values) also showed a positively skewed distribution (skewness: + 0.71) (Fig. 4, left side).

The logAUC values of the 20 subjects showed a normal distribution (mean skewness: 0.19 ± 0.87, range: −1.37 to 1.62). Also, the aggregated normalized logAUC values (Fig. 4, right side) showed a normal distribution (*p* > 0.15).

No comparable results could be located in the literature from dMD studies regarding the skewness of the distribution of intra-subject AUC values prior to log-transformation. This might be due to the type of drugs investigated in the dMD studies and/or due to the limited number of dMD probes typically used per subject.

### Factors: *site* and *leg*

**Fig. 5:**
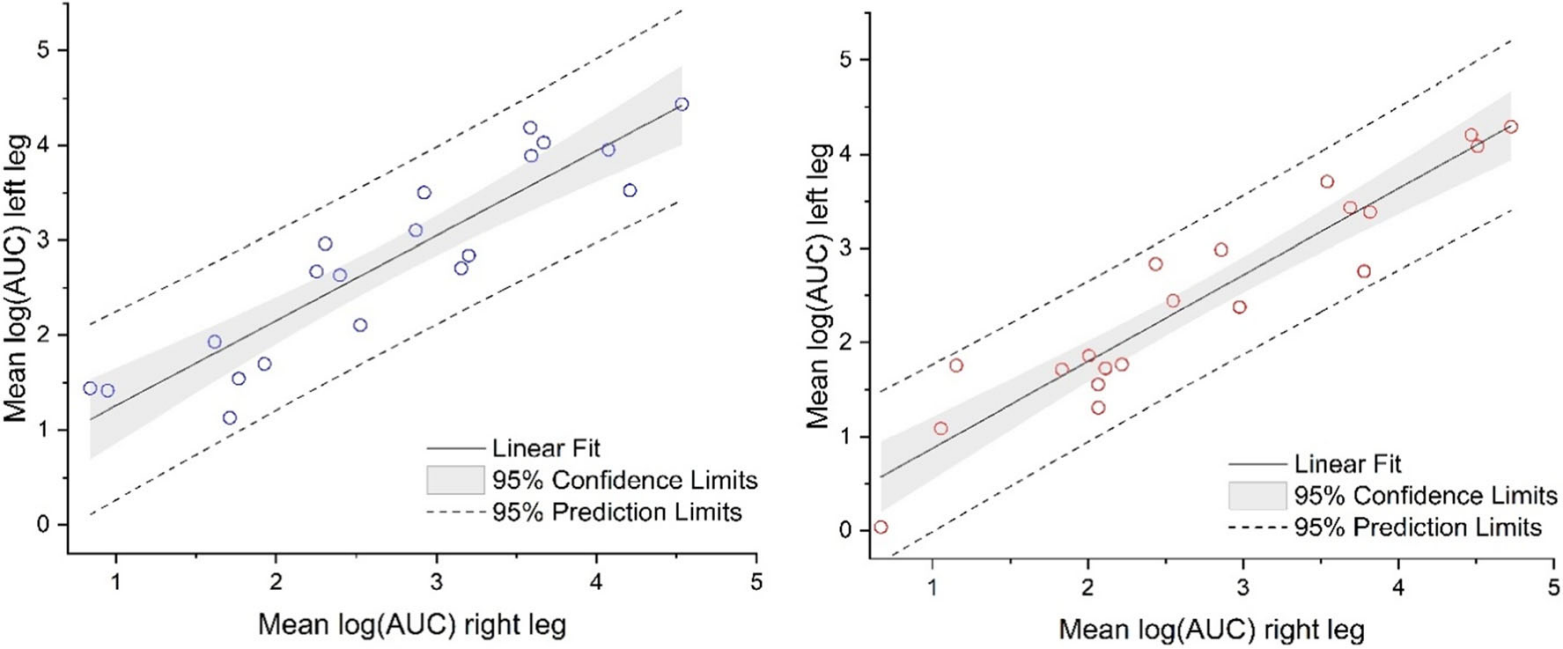
Comparison of logAUC values between treatment sites on the left and the right leg for R (left side) and T (right side)

We compared the CVs for the logAUC values of the single probes within a treatment site (45.5%), on one leg between treatment sites (49.8%), and between the legs (52.4%); the results indicated that the factor *site* (and *leg*) did not significantly contribute to the variability. The subsequent comparison of the logAUC values between the treatment sites on the left and the right leg using regression analysis confirmed good reproducibility between legs for R (r = 0.91, 2 vs. 2 treatment sites per subject) and T (r = 0.94, 1 vs. 1 treatment site per subject) (Fig. 5).

Previous dMD studies have observed variability between treatment sites on the volar forearm and have attributed them to regional differences in skin barrier function [9] or to possible differences in vasculature between distant treatment sites [12]. However, it is unlikely that local dermal vasculature or local drug clearance significantly differs from site-to-site or from probe-to-probe considering the presence of a dense capillary network of 100 capillaries/cm^2^ paralleled by lymphatic vessels in the upper dermis [20]. Benfeldt et al. applied two probes per treatment site and distinguished between the factors *site* and *probe*, but did not address potential sources of site-related variabilities [1]. Our results demonstrated that site and site-related methodological factors did not significantly contribute to intra-subject variability. As a related consideration, skin temperature reductions toward the distal portion of the extremities may contribute slightly to variability based upon our data, which indicated that temperature differences had a small (4%) contribution to variability.

### Factor: *probe*

**Fig. 6:**
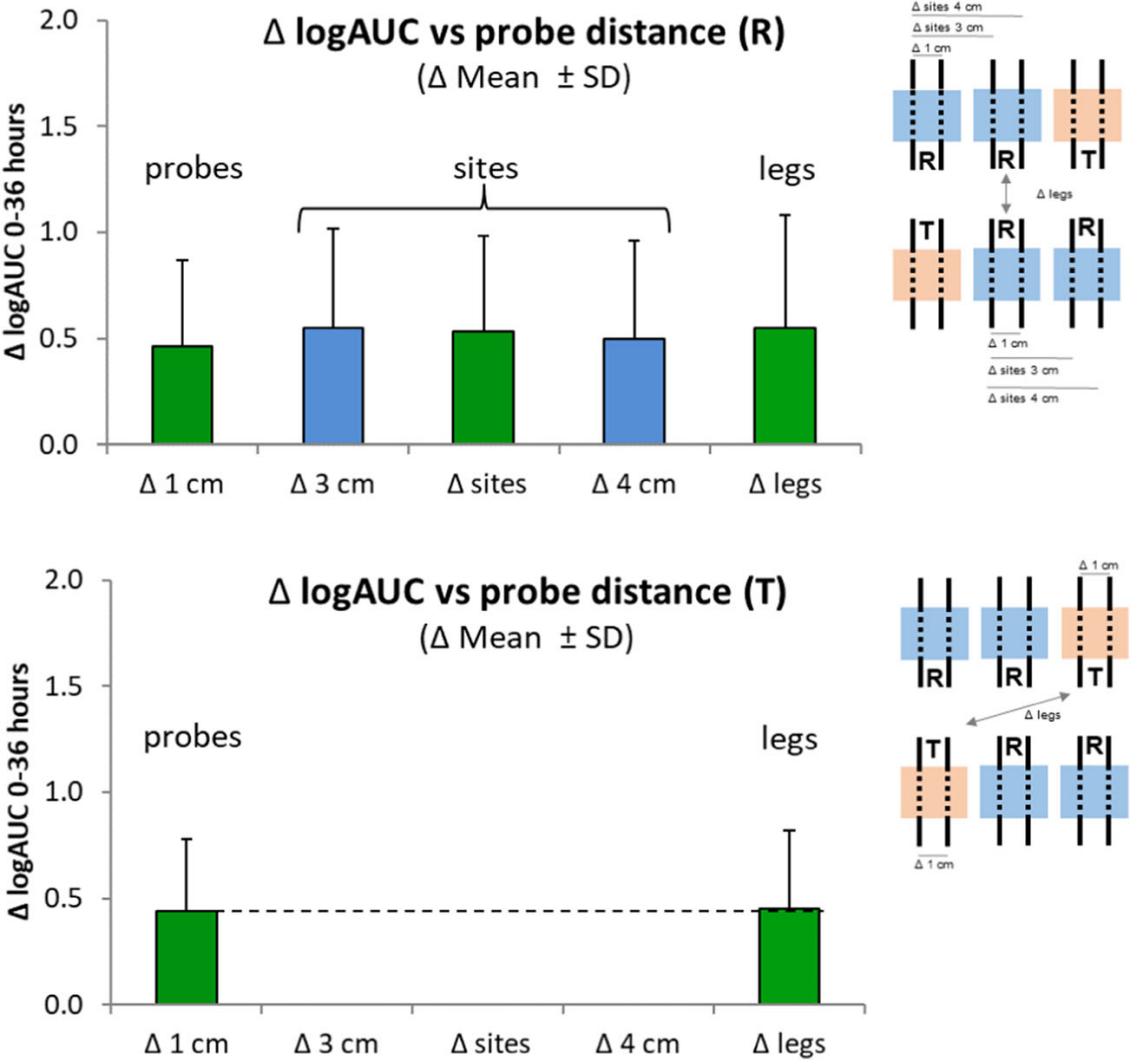
Mean differences of logAUC values between two probes depending on their positions and their distances relative to each other. Upper panel: logAUC values for R for adjacent probes in the same treatment site (Δ 1 cm) differed by logAUC 0.46 corresponding to an arithmetic mean difference of 59%. The difference between the logAUC values increased only slightly when the two probes were in two different treatment sites (Δ 3 cm and Δ 4 cm) or at different legs (Δ leg). Lower panel: logAUC values for T between adjacent probes differed by logAUC 0.44 corresponding to an arithmetic mean difference of 55%. The factor *leg* did not add any variation

To assess the contribution of the factor *probe* to the intra-subject variability*,* we first calculated the mean logAUC value-differences between (i) adjacent probes in the same treatment site (Δ 1 cm), (ii) probes in two different treatment sites on the same leg at different distances (Δ 3 cm and Δ 4 cm), (iii) probes in different treatment sites on different legs on the same subject (Δ leg) (Fig. 6). Most of the intra-subject variability was attributable to variabilities between adjacent probes.

We also investigated the co-factors (probe depth, sample volume, flow rate, recovered lactate and exchange rate of glucose) that could have been attributed to the factor *probe.* However, analyzing 80 pairs of probes from the treatment sites where R was applied revealed that none of the probe-pairs with high differences in AUC values were associated with a deviation in those co-factors. This is consistent with our results from the Pearson’s product–moment correlation analysis, which did not identify any significant probe-related co-factors. This is also in agreement with results from a dMD study performed by Kreilgaard et al. showing that data variability was not assignable to the technique itself [9]. Our intra-subject AUC values followed a log-normal distribution, which also did not implicate a methodological origin. Thus, we hypothesized that when using a hydrophilic drug with low permeation, like acyclovir, a significant portion of the observed intra-subject variability might be caused by local skin-related factors, which may influence the skin barrier function, and the drug delivery into the region of skin immediately above the probe.

While there is currently insufficient evidence to support definitive conclusions about the underlying mechanisms by which specific anatomical or structural variations in the skin may influence the rate and extent of topical drug permeation, the influence of skin-related factors on drug permeation has been studied before by In Vitro Permeation Testing (IVPT) [21–25]. Khan et al. have indicated that there is a skewed (not normal) distribution of drug penetration data which occurs when using skin instead of synthetic membranes, which might be caused by skin imperfections such as abrasions, defects or hair follicles [21]. In a large retrospective IVPT study investigating the permeability of tritiated water on skin samples, intra-subject variabilities (CVs: 38.3–115.7%) have been reported to be even higher than inter-subject variabilities (CV: 37.6%). Interestingly, the inter-subject data of this large IVPT data set followed a normal distribution. The intra-subject IVPT data, however, followed a skewed (not normal) distribution in most subjects like seen in the dOFM data [24]. Meidan et al. have attributed this behavior to the presence of local skin differences [24]. Other studies have investigated the influence of skin-related factors on drug permeation using skin with different hair follicle density [26, 27] and different in vitro models [28–32]. Results from those studies have demonstrated that hair follicles constitute a rather fast penetration pathway and that penetration via the follicular route may even be a dominant permeation pathway for hydrophilic drugs. Ogiso et al. have investigated the role of follicular penetration for acyclovir and have found a good correlation between the acyclovir flux and the hair follicle density of the skin (r = 0.666; *P* < 0.05) [26].

Assuming a hair follicle density in human skin of approximately 17 follicles/cm^2^ for men and women at the thighs [33, 34], the probability of a dOFM probe (length: 15 mm, diameter: 0.5 mm) to detect acyclovir penetrating via the follicular route from at least one follicle is more than 50%. Therefore, particularly with poorly permeating drugs, the use of more than two probes per treatment site (or per product) might be beneficial as it could further reduce variability and facilitate successful BE assessments comparing the dermal PK of a drug from T and R products in efficient BE studies with populations of approximately 20 subjects [11]. Collectively, our data and the results summarized above may suggest that the intra-subject variability (which is a determinant of the power of a dOFM BE study) may arise, at least in part, from highly localized local ‘shunts’ in the barrier that have the effect of increasing the variability in dermal PK results. The impact of this variability on a BE study may be minimized by using multiple replicate probes as well as appropriate statistical analyses, using log-transformed data for any statistical comparison. Notably, our data demonstrated that the site selected for a treatment was not a relevant source of variation.

Strengths of our study include a comprehensive data set including data on the dermal PK as well as on biological-methodological factors, which allowed the assessment of the sources of variability in dermal PK data after topical drug application and of their impact on topical BE assessments with dOFM. The head-to-head design of the dOFM study allowed for an investigation of intra-subject variability because the study design included positive and negative controls for BE, which had required multiple replicate high-resolution dOFM probes, in particular to enable the positive control, that finally allowed for an identification of the sources of variability.

Nevertheless, our study also has some limitations. We did not analyze the variability of each time point of the acyclovir concentration-time profile but, considering the fact that we wanted to understand the sources of variation in relation to a context of using dOFM to evaluate BE, we focused on the variability of the PK parameter AUC that is routinely utilized as an endpoint for BE assessments. We analyzed AUC, because AUC is assumed to have the highest informational content, being derived from multiple data points describing skin permeation, and we expected that the relative robustness of this parameter might best allow us to identify the sources of variability. Our analysis focussed on the large data set from R and did not consider potential formulation-specific factors such as skin penetration modifiers, which might exert a local action, modify the recovery, and impact the comparison between R and T products. Our expectation was that this risk may be low in future (actual) BE studies for which the T and R product components and compositions may be relatively similar. Notably, we used an Austrian acyclovir product as the T product, and it has a different composition than the R product, which is marketed in the U.S., and which was not expected to be bioequivalent to this R product. Also, skin conductance and TEWL measurements were not performed directly at the treatment sites. Instead, we analyzed an area of skin close to the treatment site, which might have been slightly different, and thereby, we may have potentially missed some correlation. Finally, our data analysis focused on the variability observed with a single drug (acyclovir), and one that is hydrophilic (unlike most topical drugs which are hydrophobic), and which exhibits very low skin permeation. As a consequence, while our results may be relevant to other hydrophilic drugs with similar penetration properties, it is not evident to what extent these results may also be relevant to relatively faster penetrating, hydrophobic topical drugs that can achieve relatively higher dermal PK concentrations. Continuing studies have been initiated to further elaborate on the sources of variability, starting with a moderately lipophilic, fast penetrating drug. Probe-to-probe data variability in the dermis was mainly observed when the drug was applied topically, but not when it was applied systemically, suggesting that our findings from acyclovir may potentially be extrapolated to more lipophilic drugs.

Our findings and conclusions should not be extrapolated to other studies, which are designed for a different purpose and use different equipment under different conditions. Our findings and conclusions, however, can most likely be extrapolated to other clinical studies performed for the purpose of comparative head-to-head topical BE assessments, which use implantable small dermal probes in combination with precision pumps for continuous sampling under highly standardized conditions.

## Conclusion

This comprehensive analysis of what is, to date, the largest dermal acyclovir data set obtained by continuous dOFM sampling, has characterized the main sources of variability in a topical dOFM BE study and provided information on the relevance of these sources of variability for topical BE assessments. The results are based upon data from a dOFM study with a hydrophilic topical drug, relatively low amounts of which permeate into the skin. Inter-subject variability dominated the overall variability, and was caused by inter-subject skin barrier differences, but due to the head-to-head study design, the inter-subject variability does not influence the BE assessment in dOFM studies.

We found a rather low intra-subject variability which is the key for a statistically powerful head-to-head BE study design. None of the methodological factors accounted for this intra-subject variability, and the characteristics of our data support the hypothesis that a significant proportion of the observed intra-subject variability in topical studies might be caused by local skin-related biological factors, e.g. hair follicles, when using a hydrophilic drug with low permeation, like acyclovir.

Additional BE studies would help to further characterize the variability of BE data for topical drugs with different physicochemical properties and/or with greater amounts permeating into the skin. The insights from the work reported here about variability in topical bioavailability and cutaneous pharmacokinetics, and about the source of this variability, directly informs the considerations for the appropriate design/setup of successful BE studies with a minimal number of subjects. The finding that sites do not contribute to variability may mean that any randomization of T and R sites is acceptable. The low intra-subject variability supports the concept of a head-to-head BE analysis of topical products (T vs R) within a relatively small number of subjects that can adequately power statistical conclusions. The inference that the variability of intra-subject data is of a biological origin linked to localized variation in skin microstructures confirms the appropriateness of using replicate probes to characterize the dermal PK of each product. Finally, the disclosure of the data characteristics reported here may prompt the exploration of alternative ways of data analyses. Thus, such a comprehensive data analysis supports the optimization of topical dOFM BE study designs, and the potential development of other efficient methods for topical BE assessments that may promote the availability of safe and effective generic topical products.

## Abbreviations

ANOVA: analysis of variance
AUC: Area under the dermal concentration-time curve
BE: bioequivalence
BMI: body mass index
C_max_: maximum dermal concentration
CV: % coefficient of variation
dMD: dermal microdialysis
dOFM: dermal open flow microperfusion
FDA: United States Food and Drug Administration
logAUC values: log-transformed AUC values
logC_max_: log-transformed C_max_ values
PK: pharmacokinetics
R: reference product
T: test product
TEWL: transepidermal water loss
U.S.: United States
